# Recommendations for Optimizing Research Regarding the Effects of Dehydration on Athletic Performance

**DOI:** 10.1007/s40279-025-02310-6

**Published:** 2025-09-03

**Authors:** Rúben Francisco, Lawrence E. Armstrong, Analiza M. Silva

**Affiliations:** 1 Exercise and Health Laboratory, CIPER, Faculdade Motricidade Humana, Universidade de Lisboa, Lisbon, Portugal; 2 Sport Sciences School of Rio Maior, Polytechnic University of Santarém, Rio Maior, Portugal; 3 Korey Stringer Institute, University of Connecticut, Storrs, CT USA

## Abstract

Dehydration’s adverse impact on athletic performance is a critical concern in sports science, yet its investigation remains challenging due to interindividual variability and methodological inconsistencies. Traditional reductionist approaches, which isolate single variables, are insufficient to capture the multifactorial nature of dehydration, which emerges from dynamic interactions among physiological and behavioral factors. These include the method of inducing dehydration, protocol duration, type of fluid loss, environmental conditions, participant characteristics, assessment techniques, and potential nocebo effects. Despite the complexity and relevance of this issue, standardized guidelines for designing sport-specific dehydration studies are lacking. This article addresses this gap by cataloging key interacting variables and offering structured, adaptable guidance rather than proposing a rigid framework. It introduces a dual framework of ‘challenges and considerations’ and ‘recommendations’ for each factor, aiming to support researchers in developing rigorous, context-specific study designs. Ultimately, this approach will promote a more nuanced understanding of dehydration and facilitate reproducible, sport-relevant research advancement.

## Key Points

**Table:** 

Reductionist approaches that isolate single variables fail to capture the complexity of dehydration, which results from dynamic interactions among physiological and behavioral factors.
Despite the relevance of dehydration to athletic performance, comprehensive research recommendations remain scarce.
This article fills the gap by identifying key interacting variables and offering structured guidance through a dual framework of ‘challenges and considerations’ and ‘recommendations’ for each factor.

## Introduction

The detrimental effects of dehydration on athletic performance have been widely debated in the scientific literature [1–5]. There is considerable consensus that dehydration can impair endurance performance, especially when fluid losses exceed 2% of body mass [3–5]. However, beyond such established findings, drawing robust and generalizable conclusions across the broader spectrum of exercise modalities remains challenging due to substantial methodological variability across studies [2, 3, 6]. This variability reflects the complex interplay of biological, behavioral, and environmental factors, such as the type and duration of dehydration, baseline hydration status, participant characteristics, performance measures, environmental conditions, blinding protocols, and assessment methods.

Such complexity impedes direct comparisons between studies and undermines the formulation of reliable, generalizable conclusions. This is a common issue across the broader field of sports science. However, it is particularly pronounced in dehydration research due to the multitude of interacting variables and the sensitivity of hydration-related outcomes to methodological differences. For instance, some studies report significant impairments in aerobic [7], muscular [8], and cognitive [9] performance due to dehydration, while others find minimal or no significant impact [10–12]. These inconsistencies may risk leading coaches, nutritionists, and athletes to underestimate dehydration’s impact, resulting in suboptimal preparation.

A potential root of these discrepancies lies in the limitations of reductionist approaches, which fail to account for dehydration’s multifactorial and dynamic nature [1–5]. A multifactorial perspective is more appropriate, as dehydration arises from the interaction of numerous physiological and behavioral variables.

Despite the relevance of dehydration to sports science and nutrition [13] and its inherent complexity [1–5], comprehensive research recommendations remain lacking. This article addresses the gap by identifying key interacting variables and providing structured guidance. It introduces a dual framework comprising ‘challenges and considerations’ and ‘recommendations’ for each factor, aiming to assist researchers in designing rigorous, context-specific studies. This approach seeks to foster a more nuanced understanding of dehydration and to facilitate reproducible, sport-relevant research advancements.

## Dehydration: The Controversial Term Without a Consensus Definition

Experts continue to debate the definition of dehydration [14]. As mentioned by the group of experts [14], dehydration is traditionally seen as excessive body water loss [15–17], while modern perspectives focus on the affected fluid compartment, especially the extracellular space, and classify dehydration as hypotonic, isotonic, or hypertonic [5, 18]. As also noted in the document prepared by the multidisciplinary group addressing the definition of dehydration [14], the Dehydration Council [19] recommends clinically oriented terms like ‘water-loss’ and ‘salt-loss’ dehydration, while European guidelines [20] choose ‘low-intake dehydration’ to highlight inadequate fluid consumption. Some other recognized authors [5] differentiate ‘extracellular dehydration’ (salt loss) from ‘intracellular dehydration’ (osmotic loss), with some proposing that ‘dehydration’ be reserved for the latter [21]. Beyond terminology, a critical distinction exists between dehydration as a process (i.e., fluid loss) and hypohydration as a water deficit state [13, 22]. In sports, dehydration typically occurs progressively during exertion, typically at a rate of 0.5–1.7 L/h, depending on exertion and environmental heat stress [23], potentially leading to hypohydration if fluids are not replenished [5].

Exercise-induced dehydration often results in a 2–5% reduction of body mass and total body water (TBW), accompanied by increased plasma osmolality (*P*_OSM_) and concentrated urine. This has led to the common but flawed assumption that concentrated urine, during ordinary daily activities with no exercise, reflects a dehydrated state. For example, non-athletes with low 24-h water intake exhibit higher arginine vasopressin (AVP) levels and relatively concentrated urine alongside lower urine volumes, without changes in body mass or *P*_OSM_ [24–26] (i.e., indicating that TBW is not diminished). In these cases, more concentrated urine likely reflects regulatory hormonal responses rather than deficits in body water content [27]. These findings suggest that habitual low fluid intake does not necessarily imply dehydration, prompting Kavouras [27] to introduce the term ‘*underhydration’*—a condition characterized by low water intake (WI) and concentrated urine without a fluid deficit. The mechanisms represent an acute response of AVP to small deficits, triggering water retention (i.e., concentrating urine) and thirst [28]. Consequently, athletes who consume less than adequate water on a given day may exhibit concentrated urine acutely, during ordinary daily activities, without reduced TBW (i.e., whole body dehydration). This phenomenon is distinct from the exercise-induced dehydration described above. A controlled study using dilution techniques in elite female and male athletes supports this paradigm. When a low 24-h WI group was compared with a high WI group, the athletes with a high WIexhibited a statistically greater urine specific-gravity (USG) but no between-group differences of extracellular, intracellular, or TBW [29].

In alignment with expert recommendations [14], the following terminology will be adopted:

- **Hypertonic dehydration** is the most prevalent form in athletic contexts, typically resulting from prolonged sweating or insufficient WI during physical exertion. It is characterized by a pure water deficit that increases extracellular fluid osmolality, subsequently drawing water out of cells and causing intracellular dehydration. This hyperosmolar state activates hypothalamic osmoreceptors, triggering AVP release and thirst to conserve body water and restore homeostasis [5].
- Although not the focus of this article, **isotonic dehydration**, defined as the proportional loss of water and sodium, merits brief mention. Common causes include diarrhea or diuretic use. Unlike hypertonic dehydration, isotonic dehydration does not raise extracellular osmolality. Baroreceptor-mediated mechanisms, including activation of the renin–angiotensin–aldosterone system (RAAS) and AVP secretion, support volume repletion [5].

**Fig. 1:**
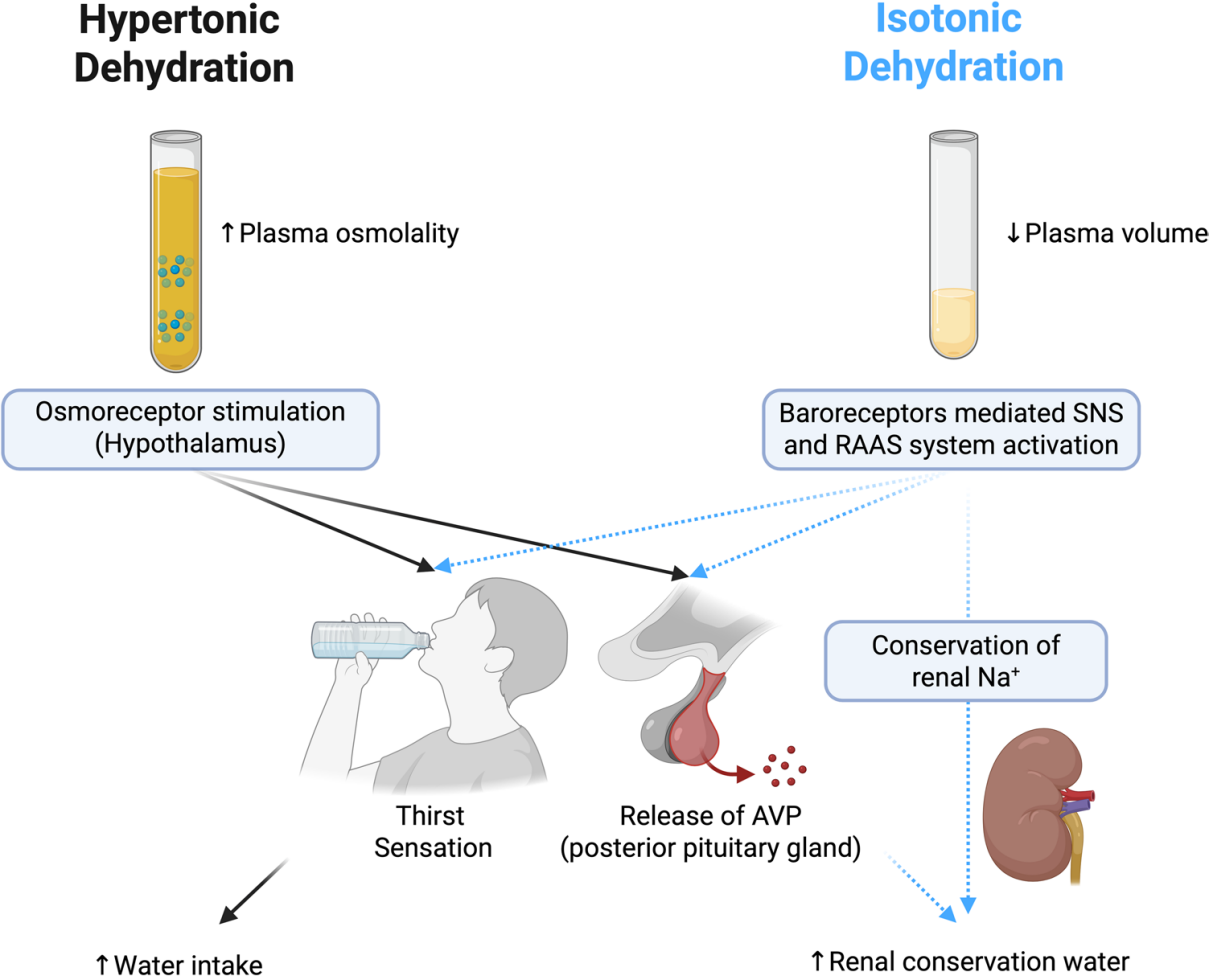
The homeostatic mechanisms underlying the two primary forms of dehydration—hypertonic and isotonic—differ in their predominant physiological triggers. Hypertonic dehydration primarily elicits an osmotic-dependent response, whereas isotonic dehydration predominantly activates a volume- and pressure-dependent response. Notably, the osmotic response exhibits greater sensitivity and is the principal regulator of water homeostasis. It is essential to recognize that both responses often occur concurrently. *AVP* arginine vasopressin, *Na*^+^ sodium, *RAAS* renin–angiotensin–aldosterone system, *SNS* sympathetic nervous system. Created in https://BioRender.com

Figure 1 illustrates hypertonic and isotonic dehydration and the key homeostatic responses that aim to restore fluid balance.

## Recommendations for Conducting Studies Testing the Effects of Dehydration on Athletic Performance

**Fig. 2:**
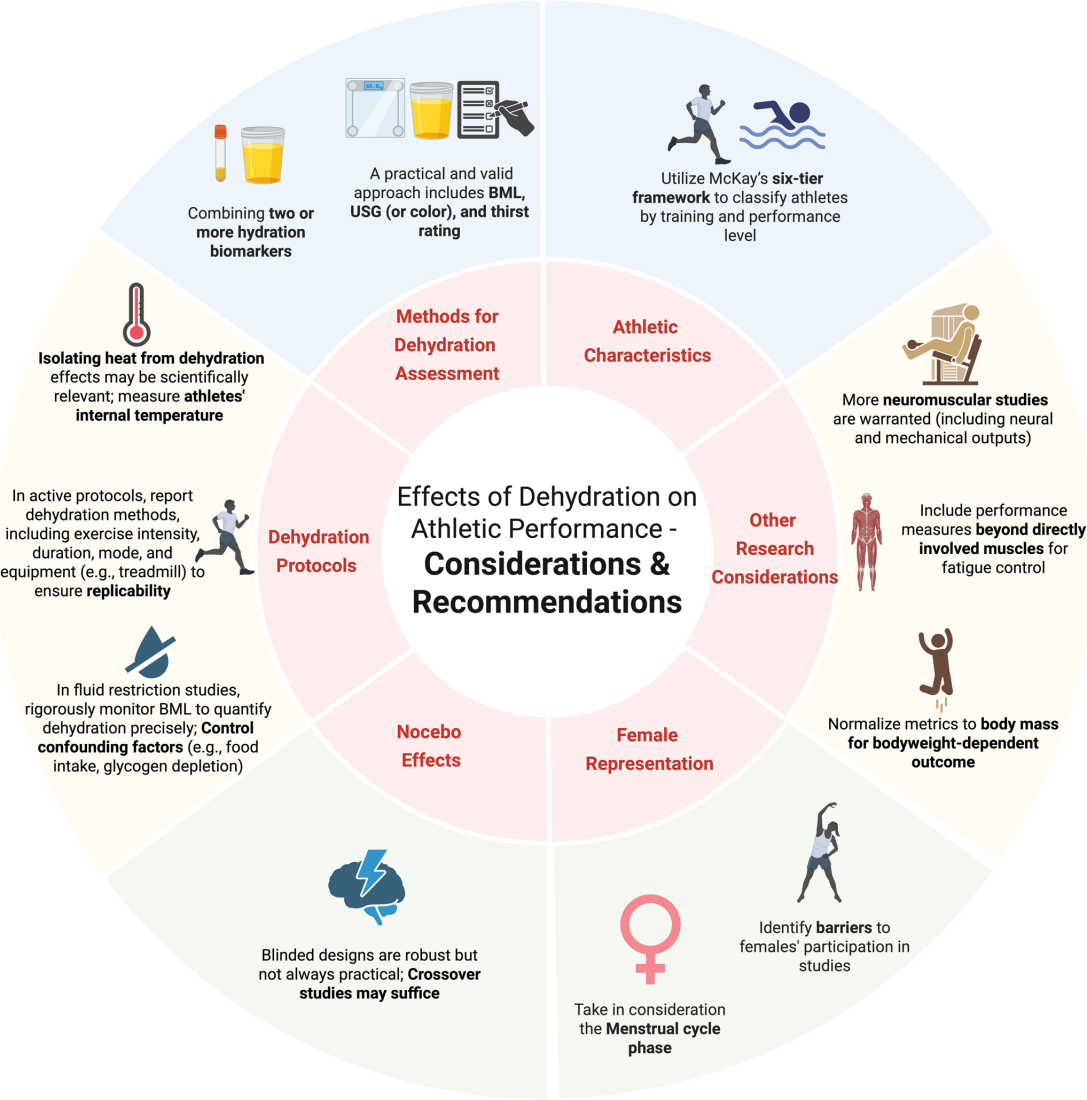
Proposed key design considerations (inner circle) and recommendations to optimize (outer circle) research regarding the effects of dehydration on athletic performance. *BML* body mass loss, *USG* urine-specific gravity. Created in https://BioRender.com

Figure 2 illustrates the factors described in Sects. 3.1 to 3.6.

### Methods for Dehydration Assessment

*Challenges and Considerations*

Defining dehydration is challenging due to its complexity and dynamic nature (see Sect. 2). Maintaining TBW relies on a complex set of homeostatic mechanisms that regulate WI, conservation, and excretion [30]. Water is distributed between intracellular and extracellular compartments, with constant shifts driven by osmotic gradients [31]. Although ion concentrations differ between the intracellular and extracellular compartments, electrochemical balance is preserved across cell membranes, allowing water to move freely in response to osmotic gradients. Within the extracellular space, fluid shifts (such as from the vascular to the interstitial space) are primarily governed by Starling forces and facilitated by lymphatic drainage through the thoracic duct [14, 32].

Considering the complex dynamics of dehydration, no single hydration biomarker is universally applicable, as each is influenced by factors such as circadian rhythms, dietary intake, and environmental conditions [33–36]. Although numerous techniques exist, those most commonly employed in athletic settings include (i) TBW and extracellular water measured via isotope dilution (intracellular water determined by subtracting extracellular water from TBW) [37–39] or estimated by bioelectrical impedance analysis [40]; (ii) plasma markers such as osmolality, sodium concentration, hematocrit, and regulatory hormones (e.g., AVP/copeptin) [41]; (iii) urinary markers, including osmolality, USG, and color (41); (iv) changes in body mass; and (v) other variables such as salivary and tear osmolality or clinical signs and symptoms [41, 42]. The variability in methodology, often insufficiently described, complicates data interpretation across studies, underscoring the need for standardized protocols [42].

*Recommendations*

1. The first recommendation is that researchers avoid using one method to assess dehydration in repeated measurements [42, 43]. Combining at least two hydration biomarkers for a reliable assessment of fluid intake adequacy is strongly recommended. Three indices combined provide even greater accuracy in identifying dehydration or inadequate fluid intake [41, 43].
2. If researchers have limited resources, the WUT (weight, urine, thirst) approach offers a practical, rapid, and valid method for assessing hydration as supported by the 2022 International Olympic Committee consensus [44]. It includes (i) body mass change (> 1–2% indicates dehydration), (ii) urine specific gravity (USG > 1.020 or color > 5 arbitrary units), and (iii) subjective thirst rating [45]. While no single marker is definitive, two positive indicators suggest likely insufficient fluid intake, and all three strongly indicate inadequacy [45]. However, accuracy may be reduced post-exercise (due to delayed thirst), in the early morning (due to concentrated urine), or with factors like sodium imbalance, recent intake, or unrelated body mass changes.
3. When measuring acute dehydration via body mass, a scale measuring to the nearest 10 g should be used to ensure greater precision. Small fluctuations in body mass are critical for accurately assessing the degree of dehydration.
4. Regarding urine assessments, in situations where researchers aim to assess athletes with a chronic low WI, it has been observed that USG values and urine osmolality (> 700 mmol/kg identifies dehydration) may be higher in low drinkers than in high drinkers, despite similar body water volumes [29]. This reinforces that urine alone may not serve as an indicator of dehydration, but of low WI. Combining urine measurements with other biomarkers is highly recommended.
5. If available, *P*_OSM_ (> 290 mmol/kg) may be used as a diagnostic parameter [44]. Some laboratories consider P_OSM_ the gold standard, but it has notable limitations [46]. It remains stable due to the tightly regulated *P*_OSM_-AVP loop, making it suitable for detecting acute changes but less effective for identifying mild or chronic dehydration, such as that from a regular low WI in athletes [47–51]. Additionally, *P*_OSM_ may not respond reliably to rehydration post-exercise, shows poor correlation with body mass loss in some scenarios, and demonstrates high inter-individual variability for similar body water changes [46]. Its use alone can thus be misleading. Additionally, it is technically complex, costly, and time-consuming. For further details, see the reference [46].
6. In laboratory-based research, additional blood measures can offer valuable mechanistic insights into dehydration. These include plasma volume changes (e.g., via hematocrit; > 2% changes indicate dehydration) and fluid-regulatory hormones such as vasopressin or copeptin (no cut-off identified) [52].
7. Saliva is a promising tool for assessing pre- and post-dehydration status in athletes during exercise [41, 53]; however, its reliability is limited to controlled conditions without confounders. Saliva is influenced by food and fluid consumption and requires costly, specialized analysis [54].
8. Bioelectrical impedance analysis can assess water compartments (the reference method is dilution techniques) via predictive equations [55] and allow the interpretation of the raw parameters [37–39]—resistance, reactance, capacitance, phase angle, and impedance—providing insight into soft tissue hydration [56]. Bioelectrical impedance vector analysis (BIVA) refines this by evaluating vector patterns without predictive models [57]. Although practical and relatively low cost, BIA requires specialized equipment, including analyzers and electrodes.

### Athletic Characteristics

*Challenges and Considerations*

A major challenge in dehydration research is the inconsistent classification of athletes, with vague terms like ‘elite’ or ‘recreational’ often lacking clear criteria [58]. This hampers study comparability and obscures differences in dehydration responses linked to training status and sport-specific demands. Athletes’ responses vary based on training history, experience, recovery capacity, and physiological adaptation. For instance, highly trained athletes often exhibit more efficient thermoregulation [59], such as earlier sweating and greater sweat rates, than recreational individuals [60]. Comparisons must be made cautiously, as studies involving recreational athletes may not be generalizable to elite populations and vice versa [60]. Poor classification of participants and limited ecological validity reduce the applicability of findings to real-world performance. Clearly defining athlete types and training levels is essential for understanding dehydration’s impact on performance and for developing effective hydration strategies.

*Recommendations*

1. To address these challenges, we refer to McKay’s framework [58], which classifies participants by training and performance levels for a more straightforward interpretation of dehydration effects. The six-tier system includes Tier 0 (sedentary individuals), Tier 1 (recreationally active individuals without structured training), Tier 2 (regularly trained individuals aiming for local competitions), Tier 3 (highly trained national-level athletes), Tier 4 (elite international competitors), and Tier 5 (world-class athletes setting records). A full review of the original study is recommended [58].

### Female Representation

*Challenges and Considerations*

Female representation remains a critical issue. In a new era where organizations like the National Olympic Committees seek to implement policies that promote gender balance in sports and equitable representation at the Olympic Games [61], understanding women’s physiological responses to dehydration is essential, yet research is limited [6]. Attention must be paid to the role of female hormones (i.e., estrogen and progesterone) that may influence fluid balance, thermoregulation, internal body temperature, sweating thresholds, and renal sodium handling [62]. This is particularly relevant given the persistent underrepresentation of female athletes in research, where male data often serves as a proxy [63]. It is worth noting that female athletes may exhibit subclinical disturbances, such as luteal phase defects due to luteinizing hormone insufficiency, leading to reduced progesterone levels regardless of an apparent regular cycle [64]. Moreover, menstrual disorders like oligomenorrhea and amenorrhea are common in this population [65]. Thus, while hormone fluctuations may be attenuated compared with sedentary women, hormonal dysfunction can still occur, underscoring the need for context-specific investigation that accounts for menstrual status and training background.

*Recommendations*

1. Researchers should identify the primary barriers to women's participation in scientific studies and develop tailored recruitment strategies to effectively address and overcome these obstacles [66].
2. Researchers should consider the menstrual cycle in female athletes [67], as its impact on performance remains uncertain due to conflicting findings [67].
3. Research aiming to isolate hormonal effects may benefit from testing participants in well-defined menstrual phases (e.g., early follicular or mid-luteal), whereas studies seeking ecological validity may include participants without phase restrictions but should still report menstrual status and account for it analytically when feasible.

### Dehydration Protocols

The study of dehydration and its impact on athletic performance necessitates controlled dehydration protocols to achieve precise levels of fluid loss. These protocols are generally categorized into three types: (i) active dehydration, which typically involves physical exercise; (ii) passive dehydration, which usually includes methods such as heat exposure (e.g., sauna); and (iii) fluid restriction, which generally includes experimental methods that induce dehydration by limiting fluid intake over a specified period, typically 12–24 h. Each approach has distinct implications for study outcomes and challenges.

#### Active Dehydration Protocols

*Challenges and Considerations*

Active dehydration involves inducing fluid loss through exercise under controlled environmental conditions, often with limited or no fluid replacement [1]. The exercise intensity employed in these protocols can vary widely, from low-intensity steady-state activities to high-intensity interval or endurance exercises. The intensity directly affects the rate of fluid loss, as higher intensities generate more significant metabolic heat and sweating rates, leading to accelerated dehydration [23]. Studies employing active dehydration often face complex interactions such as exercise-induced fatigue or hyperthermia, which can independently influence performance outcomes [6]. Another critical aspect of the active protocols is their specificity. Dehydration protocols often employ generalized methods, such as cycling and running, regardless of the athletes’ primary sport. While these approaches can effectively induce fluid loss, they fail to account for sport-specific physiological demands, such as movement mechanics, muscle group utilization, and energy system contributions.

*Recommendations*

1. Researchers conducting active dehydration protocols should report the methods used, including detailed exercise parameters, such as intensity (e.g., percentage of maximal oxygen consumption), duration, mode (e.g., cycling, running), and equipment (e.g., treadmill), to ensure replicability by other investigators and clarify the relationship between dehydration and performance.
2. For a rigorous control of exercise intensity during dehydration, we recommend determining exercise intensity based on the percentage of the ventilatory thresholds. This method offers a more personalized and physiologically meaningful approach [68, 69]. In contrast, measures like the percentage of maximal oxygen consumption or the percentage of heart rate are less individualized. Although this method requires a graded exercise test, it improves precision and enables cross-study comparisons.
3. In mechanistic protocols, a practical approach is to implement a dehydration protocol at an intensity that minimizes exercise-induced fatigue. This strategy aligns with that of Francisco and colleagues [1], who induced dehydration through exercise below the first ventilatory threshold—the intensity at which lactate starts accumulating, and ventilation rises disproportionately to oxygen uptake. The first ventilatory threshold precedes the second ventilatory threshold, which marks a shift to predominantly anaerobic metabolism.
4. For applied or sport-specific investigations, researchers should design protocols that replicate typical training or competitive conditions, even when these involve high-intensity efforts and account for physiological adaptations unique to each sport. This may involve stratifying participants based on their athletic level (see Sect. 3.2) or heat exposure history, or controlling for these variables in the study design and analysis to accurately interpret the physiological responses and performance outcomes.
5. Active (and passive) dehydration protocols should monitor internal temperature to ensure participant safety and reduce the risk of hyperthermia. This can be done using rectal thermistors, which measure temperature in the rectum, or gastrointestinal pills, which assess temperature along the gastrointestinal tract, with the latter offering greater comfort and user acceptance [70]. However, both methods may respond more slowly to rapid internal temperature changes compared with esophageal thermometry, which provides faster readings due to its proximity to the heart and major vessels [71]. While non-invasive methods (e.g., skin sensors, tympanic or temporal artery thermometers) are practical, they remain insufficiently accurate [72, 73]. For example, the forehead thermometer strips and auditory ear canal (aural) infrared thermometers do not represent the temperature of deep internal organs [74]. While true tympanic temperature, measured by inserting a thermocouple in contact with the tympanic membrane and sealing the ear canal can closely track esophageal temperature, it carries a risk of membrane trauma and is rarely practical in field settings [75, 76].

#### Passive Dehydration Protocols

*Challenges and Considerations*

Passive dehydration protocols induce fluid loss without physical exertion, typically through controlled heat exposure in saunas or heat chambers [77]. Passive protocols minimize the confounding effects of exercise-related fatigue, enabling a more direct examination of the physiological effects of dehydration. At the same time, it raises another issue: heat exposure [78]. Heat exposure is commonly used in athletes to accelerate dehydration, with or without exercise [77, 79], and is effective in inducing fluid loss for both research and practical purposes. However, it introduces complex physiological stressors—cardiovascular strain, metabolic changes, and thermal discomfort [80]—that can independently affect performance [78]. Notably, passive heating often induces more pronounced hyperventilation than active heating [81, 82], which can reduce cerebral blood flow, increase sensations of breathlessness, and in some cases lead to peripheral symptoms such as finger tetany. These additional factors may confound the isolation of dehydration’s specific impact on athletic performance. Furthermore, because passive dehydration typically involves lower metabolic and thermoregulatory demands than active dehydration protocols, aside from combat sports that frequently employ these strategies [83], its ecological validity in some athletic performance contexts may be limited. It is important to note that diuretics would fall under the category of passive dehydration protocols; however, they are currently banned both in and out of competition as outlined by the World Anti-Doping Agency and are therefore not discussed in this article.

*Recommendations*

1. In passive protocols, the environmental conditions should be well documented, including the temperature, humidity, and wind speed.
2. In studies with heat exposure, approaches that are similar to those employed by Périard and colleagues [84] and van den Heuvel and colleagues [85] may be helpful. These studies used controlled temperature and humidity settings to isolate dehydration effects, distinguishing them from heat stress. While this separation has limited practical relevance, it offers scientific value in clarifying distinct physiological responses.
3. Internal temperature should be monitored to ensure participant safety and reduce the risk of hyperthermia during dehydration procedures, as detailed previously in Recommendation 5 of Sect. 3.4.1.
4. Furthermore, blood pressure monitoring is also advisable, particularly in light of the cardiovascular demands and potential for syncope due to peripheral vasodilation under heat stress [42].

#### Fluid Restriction Protocols

*Challenges and Considerations*

Dehydration can be accomplished by restricting fluids and foods that have high water content. Common in sports with weight classes (e.g., combat sports), this method helps athletes ‘make weight’ before competition [51, 83]. Athletes may manipulate glycogen stores by combining reduced carbohydrate intake with intense training or maintaining intake while increasing training volume. Stored in the liver and muscles, glycogen binds water at a 1:2.7 ratio [86]; thus, its reduction, through carbohydrate restriction or exhaustive exercise, lowers intracellular water content [87, 88].

Additionally, some protocols involve avoiding foods high in water content (e.g., fruits, vegetables, soups) or extending food and fluid restriction over 12–72 h [51]. While effective for acute weight loss, these practices can impair cognitive and physical performance and pose health risks when used repeatedly.

*Recommendations*

1. Specify the methods used to induce dehydration, including the duration and type of fluid and/or food restriction. If exercise is employed to achieve a higher glycogen depletion, the mode and intensity of exercise should be reported (see Sect. 3.4.1).
2. Design protocols that mimic real-world scenarios, such as pre-competition dehydration strategies or fasting before specific events.
3. Researchers should understand that caloric restriction affects energy availability, glycogen stores, and metabolic pathways [87, 88]; therefore, isolating the effects of dehydration alone becomes difficult. However, combined restrictions replicate real-world scenarios where athletes encounter simultaneous challenges in fluid, energy, and exercise (e.g., during long-distance running or combat sports).

### Nocebo Effects of Dehydration

*Challenges and Considerations*

Blinded studies are commonly used in sports science to reduce bias and control for placebo and nocebo effects. In dehydration research, some studies have successfully blinded participants to their hydration status using intravenous or intragastric fluid delivery [11, 12, 89–92]. Findings have varied: intravenous rehydration often shows no performance impairment [11, 12], while intragastric methods, using body-temperature water to avoid detection, consistently report performance declines of 8–11% [90, 91]. These differences found in performance among those studies may have originated from methodological variations [3]. Intravenous delivery bypasses the gastrointestinal tract and uses isotonic saline, potentially masking hydration cues by raising serum osmolality in both groups (euhydrated vs dehydrated). In contrast, intragastric studies use water to preserve natural hydration signals and mimic oral rehydration more closely. Additionally, these studies include small oral fluid volumes to activate oropharyngeal receptors, which may be crucial for fluid regulation [93].

As this research area is still emerging, more studies are needed to clarify dehydration’s impact on performance, and current recommendations remain preliminary.

*Recommendations*

1. The first recommendation is to expand blinded studies beyond endurance cycling exercise, as current research is limited and still emerging. Further investigation, particularly into neuromuscular function, is essential to understand dehydration’s effects fully.
2. All previous studies have been conducted in heated conditions. Investigating this phenomenon in cooler environments is crucial to determine whether environmental factors influence performance outcomes.
3. While blinded designs may be a robust methodology for investigating the effects of dehydration on performance, they are not always practical. In some cases, alternative designs, such as crossover studies, which effectively control for inter-individual variability, are preferred. However, when crossover designs are infeasible, well-controlled parallel designs using randomization and stratification can still provide meaningful insights.
4. When crossover designs are not feasible, randomly assigning participants to euhydration or dehydration protocols while ensuring stratification for key variables like age, sex, athleticism level (see Sect. 3.2), and baseline hydration status can enhance the study’s internal validity and mitigate potential confounding factors.

### Other Research Considerations

*Challenges and Considerations*

The effects of dehydration on endurance are well documented [4, 5], but its impact on neuromuscular function remains less explored. Dehydration can impair motor control and muscle output [6], though it is often unclear whether these deficits arise from neural or muscular origins [6, 8]. Elevated internal temperature may further exacerbate neuromuscular impairments, complicating experimental control [8, 78].

In athletic contexts, distinguishing dehydration-induced from exercise-induced fatigue is challenging, as exertion can confound the results [6]. Another critical factor is how dehydration impacts bodyweight-dependent performance tests (e.g., squat jumps) [94], where weight loss from dehydration may falsely appear as performance improvement if not accounted for.

Another concern is exercise-associated hyponatremia, which occurs when fluid intake exceeds sweat loss during prolonged exercise (e.g., in studies where there may be ad libitum rehydration) [95]. This can dilute blood sodium, impair performance, and cause serious complications, including edema, seizures, or death [13]. Symptoms include confusion, nausea, vomiting, and diluted urine [96].

Finally, research has attempted to define dehydration thresholds for performance decline, often identifying a ~ 2% body mass loss as critical [34]. However, most studies use binary comparisons (euhydrated vs dehydrated) and lack dose–response designs, limiting understanding of graded effects. Significant individual variability also exists—some athletes show impairments with minimal fluid loss [97], while others tolerate greater losses [59].

*Recommendations*

1. Historically, the literature has predominantly focused on endurance performance; future studies should include other modalities, such as neuromuscular performance.
2. In neuromuscular-based studies, using exercise-based dehydration protocols, researchers should assess muscle groups not primarily involved in the dehydration protocol. For example, if the dehydration involves mainly the lower limb muscles (e.g., running), incorporating upper body strength tests can enhance the study’s conclusions. However, a more ecological approach may be appropriate if the goal is to examine the combined effects of dehydration and exercise-induced fatigue [59].
3. Force production depends on both mechanical and neural factors [6]. To discern the origins of neuromuscular fatigue, studies should be designed to measure both mechanical and neural output (e.g., maximal voluntary contraction in combination with peripheral nerve stimulation) [79, 98].
4. In endurance-based performance studies, dehydration may impair output via thermoregulatory strain and cardiovascular instability rather than through dehydration alone [4]. To enhance mechanistic interpretation, researchers are strongly encouraged to include physiological assessments such as internal temperature, heart rate, and blood pressure.
5. When studies involve bodyweight-dependent outcomes, researchers should normalize performance metrics to body mass changes for tests such as squat jumps, to prevent misinterpretation due to weight reduction alone [94].
6. To reduce the risk of exercise-associated hyponatremia [95] in studies that include fluid intake during dehydration, researchers must ensure that athletes do not start exercising with low USG (< 1.005) [99, 100], monitor for early symptoms, and limit athletes’ fluid intake during endurance events to under 700 mL/h [34, 101].
7. A dose–response approach [97] enables the assessment of graded dehydration levels as a continuous variable, moving beyond the conventional 2% body mass loss threshold commonly cited [3–5] as imparting endurance performance. This approach allows for robust internal controls and facilitates a more comprehensive understanding of the nuanced, context-specific, and individualized responses to dehydration across different exercise modalities, environmental conditions, and populations.

## Summary and Conclusions

Although the adverse effects of dehydration on athletic performance are well established, definitive conclusions remain elusive due to substantial variability in experimental design. The complex and dynamic nature of human fluid balance makes dehydration difficult to define and assess, with methodological and physiological factors further complicating interpretation.

This article highlights key sources of variability, including assessment methods, athlete characteristics, underrepresentation of females, dehydration protocols, and possible nocebo effects. These are summarized in Fig. 2, which presents core considerations (inner circle) and corresponding recommendations (outer circle).

To address these challenges, we proposed a structured framework of challenges and considerations alongside recommendations to promote greater standardization and rigor in dehydration research.

## Abbreviations

*P*_OSM_: Plasma osmolality
AVP: Arginine vasopressin
USG: Urine specific gravity
WI: Water intake
TBW: Total body water
